# Associations of Poincaré Plot-Derived Parameters with Heart Rate Variability and Autonomic Reflex Testing in a Real-World Clinical Population

**DOI:** 10.3390/diagnostics16071016

**Published:** 2026-03-27

**Authors:** Branislav Milovanović, Nikola Marković, Maša Petrović, Aleksa Korugić, Milovan Bojić

**Affiliations:** 1 Institute for Cardiovascular Disease “Dedinje”, 11000 Belgrade, Serbia; 2 School of Medicine, University of Belgrade, 11000 Belgrade, Serbia; 3 Clinic for Otorhinolaryngology and Maxillofacial Surgery, University Clinical Center of Serbia, Serbia Pasterova 2, 11129 Belgrade, Serbia

**Keywords:** Poincaré plot, heart rate variability, autonomic nervous system, nonlinear analysis, dysautonomia

## Abstract

**Background/Objectives:** Poincaré plot analysis represents a nonlinear approach to heart rate variability (HRV) assessment, but the physiological meaning of several derived parameters remains unclear. This study aimed to evaluate associations between selected Poincaré plot-derived parameters, conventional HRV indices, and cardiovascular autonomic reflex tests in a real-world clinical population. **Methods:** This observational study included 269 adult patients referred for evaluation of suspected autonomic dysfunction. All participants underwent short-term resting ECG, cardiovascular autonomic reflex testing, and 24 h Holter ECG monitoring. Poincaré plot-derived parameters were analyzed in relation to short- and long-term HRV measures using the Spearman correlation with false discovery rate correction, and group comparisons were performed based on reflex test results. **Results:** Several Poincaré plot-derived parameters showed strong correlations with long-term HRV indices. VLI and LA were primarily associated with global and long-term autonomic variability, whereas VAI and SA were more closely related to parasympathetic modulation. Associations with short-term HRV were generally weak. Lower values of selected parameters were observed in patients with abnormal parasympathetic reflex tests, while no significant differences were found in relation to orthostatic hypotension. **Conclusions:** Poincaré plot-derived parameters capture complementary aspects of autonomic regulation beyond conventional HRV indices and may enhance autonomic phenotyping in clinical settings.

## 1. Introduction

The autonomic nervous system (ANS) plays a central role in the involuntary regulation of most non-somatic end organs and is essential for adaptation to internal and external stressors, thereby maintaining physiological homeostasis [1]. ANS dysfunction may arise from primary disorders directly affecting the ANS (primary autonomic neuropathies) or secondarily as a consequence of diseases involving other organ systems, most notably cardiovascular diseases and diabetes mellitus. Importantly, these conditions exhibit a bidirectional interaction with autonomic regulation, whereby autonomic imbalance both contributes to and results from systemic pathology [1,2,3,4,5].

Diagnostic approaches to ANS assessment range from respiratory and orthostatic reflex tests to advanced electrocardiographic analyses, including heart rate variability (HRV), deceleration capacity (DC), heart rate turbulence (HRT), and baroreflex sensitivity (BRS) [6,7,8,9,10,11]. Among these methods, HRV—particularly time- and frequency-domain measures—is most widely used and has demonstrated robust prognostic value across a spectrum of cardiovascular diseases [9,12,13,14,15].

Beyond conventional linear ECG-derived parameters, nonlinear analytical techniques have increasingly been employed to capture the complex dynamics of cardiac rhythm regulation [7]. Nonlinearity reflects the intrinsic complexity and unpredictability of heart rate fluctuations that cannot be adequately described by linear models and arises from the interplay of multiple regulatory mechanisms governing cardiac autonomic modulation [7]. One of the most widely studied nonlinear methods is the Poincaré plot, which graphically represents the relationship between consecutive RR intervals in a two-dimensional phase-space format [16,17]. Typically, the RR interval Poincaré plot forms an elongated cluster of points along the line of identity; dispersion perpendicular to this line reflects short-term variability, whereas dispersion along the line corresponds to long-term variability [16].

The most extensively investigated Poincaré plot-derived parameters are SD1, SD2, and their ratio. SD1 primarily reflects short-term HRV and parasympathetic modulation, while SD2 represents long-term variability influenced by both autonomic branches, with stronger associations with sympathetic indices [7,16,17,18,19]. Consequently, the SD2/SD1 ratio has been proposed as a geometric surrogate marker of sympathovagal balance [19].

**Figure 1:**
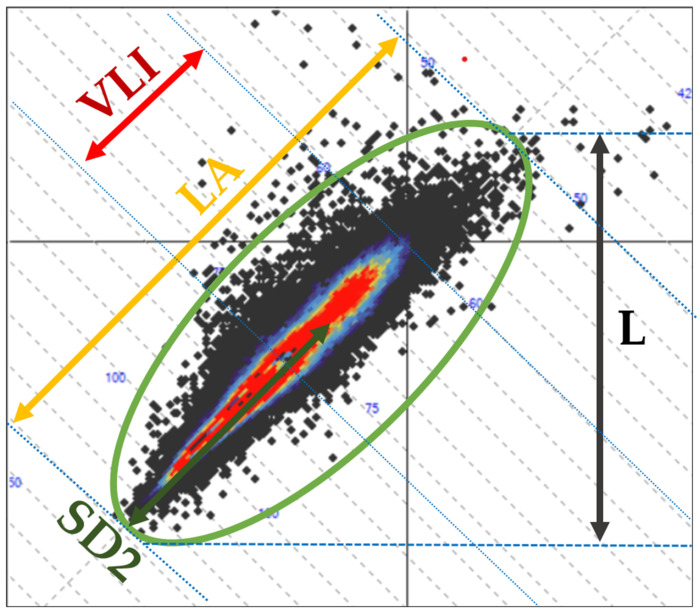
Graphical representation of selected Poincaré plot-derived parameters illustrating their distribution and geometric characteristics. Arrows indicate: red—VLI, yellow—LA, green—SD2, and gray—L. VLI—vector length index; LA—Poincaré long axis; SD2—long-term variability; L—Poincaré length.

**Figure 2:**
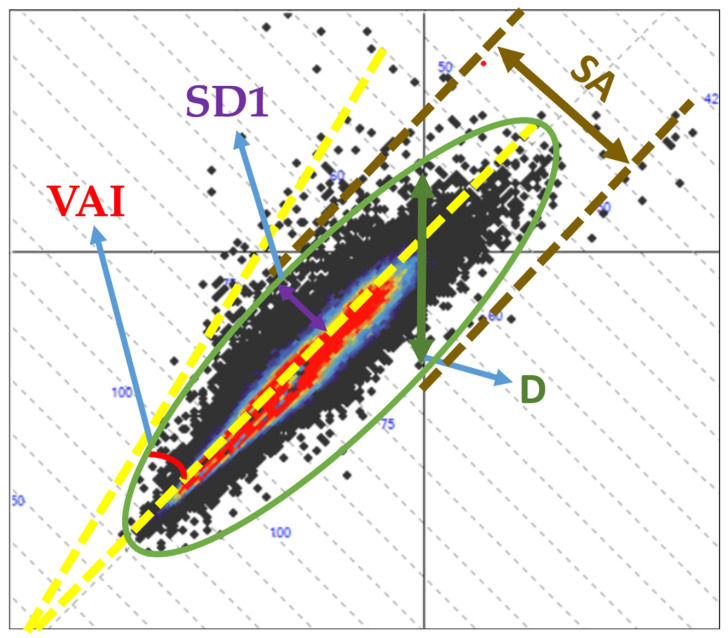
Graphical representation of additional Poincaré plot-derived parameters illustrating their distribution and geometric characteristics. Arrows indicate: red—VAI, purple—SD1, brown—SA and green—D. VAI—vector angle index; SD1—short-term variability; SA, Poincaré short axis; D, Poincaré dispersion.

In addition to SD1, SD2, and their ratio, 24 h Holter ECG recordings may yield additional Poincaré plot-derived parameters, including the vector length index (VLI), Poincaré length (L), Poincaré dispersion (D), long axis (LA), short axis (SA), and vector angle index (VAI). All parameters except VAI are expressed in milliseconds, whereas VAI is expressed in degrees (Figure 1 and Figure 2). Despite their availability in certain Holter analysis systems, the physiological underpinnings and clinical significance of these parameters remain insufficiently characterized. To date, only one study has reported that VLI and VAI demonstrate superior discriminative performance compared with traditional HRV indices, with reduced parasympathetic activity and increased sympathetic predominance being associated with lower values of both parameters [20]. Given the well-established role of ECG-based metrics in risk stratification and disease prediction, further characterization of these less-explored Poincaré plot-derived parameters may hold clinical relevance.

The aim of this study is to enhance understanding of less-well-defined Poincaré plot-derived parameters (VLI, VAI, L, D, LA, and SA) in a heterogeneous clinical population comprising individuals with cardiovascular and non-cardiovascular conditions. Specifically, we aim to: (1) analyze inter-parameter correlations; (2) examine associations with long-term HRV parameters derived from 24 h Holter ECG recordings; (3) assess correlations with short-term HRV indices; and (4) evaluate differences in parameter values between groups with normal and abnormal findings on selected cardiovascular reflex tests.

## 2. Materials and Methods

### 2.1. Study Population

This observational study included 269 consecutive adult patients (≥18 years) referred to the Neurocardiology Laboratory of the Cardiology Clinic, Institute for Cardiovascular Diseases “Dedinje”, between January 2023 and December 2025, for evaluation of suspected ANS dysfunction. Referrals were prompted by a broad spectrum of cardiovascular and non-cardiovascular conditions and symptoms suggestive of dysautonomia, including syncope, orthostatic intolerance or hypotension, chronic fatigue, palpitations, dizziness, cognitive complaints, and exercise intolerance.

The study cohort represents a heterogeneous, real-world clinical population comprising patients with arterial hypertension, coronary artery disease, diabetes mellitus, thyroid disorders, chronic fatigue syndrome, post-infectious conditions, Lyme disease, and other disorders frequently associated with autonomic dysfunction. Demographic and clinical characteristics of the study population are summarized in Table 1.

Exclusion criteria included known primary autonomic failure syndromes (e.g., pure autonomic failure, multiple system atrophy, and Parkinson’s disease), absence of sinus rhythm during ECG recordings (including atrial fibrillation or other sustained arrhythmias), presence of implanted cardiac pacing or defibrillation devices, hemodynamically significant structural heart disease (e.g., severe aortic stenosis or left ventricular outflow tract obstruction), advanced respiratory or renal failure, or any condition precluding reliable HRV analysis.

### 2.2. Ethical Approval

The study protocol was approved by the Ethics Committee of the Institute for Cardiovascular Diseases “Dedinje” (Approval No. 6472, issued 11 December 2024). The study was supported by grant 451-03-68/2020-14/200156 from the Ministry of Education, Science, and Technological Development of the Republic of Serbia and by the COVANSA grant from the Science Fund of the Republic of Serbia. All procedures were conducted in accordance with the principles of the Declaration of Helsinki.

### 2.3. Study Protocol

All participants underwent a standardized autonomic assessment performed as part of routine clinical evaluation. The protocol consisted of a short-term (5 min) resting ECG recording in a supine position, followed by cardiovascular autonomic reflex testing (CART) and 24 h Holter ECG monitoring for long-term HRV analysis. All examinations were performed according to standardized laboratory procedures.

#### 2.3.1. Cardiovascular Autonomic Reflex Tests (CARTs)

Cardiovascular autonomic reflex tests were performed to evaluate both branches of the autonomic nervous system [6]. Parasympathetic function was assessed using the Valsalva maneuver (VM), heart rate response to deep breathing (HRB), and heart rate response to standing. Sympathetic function was evaluated using the sustained handgrip test and assessment of orthostatic hypotension (OH).

Test results were classified as normal, borderline, or abnormal according to established criteria [6]. In accordance with the recommendations of Bellavere et al., borderline results were considered to reflect technical variability and were excluded from comparative group analyses, which were limited to normal and abnormal findings [21]. Due to the very small number of patients with normal results on the handgrip test and heart rate response to standing, these tests were not included in comparative analyses.

#### 2.3.2. Short-Term ECG and HRV Analysis

Short-term ECG recordings were obtained using a standard 12-lead ECG system, and HRV was analyzed using dedicated software. Time-domain HRV indices included SDNN, defined as the standard deviation of normal-to-normal RR intervals; RMSSD, reflecting the root mean square of successive RR interval differences; pNN50, representing the percentage of adjacent RR intervals differing by more than 50 ms; and the HRV triangular index, a geometric measure of overall HRV. Frequency-domain analysis included total power (TP); very-low-frequency (VLF), low-frequency (LF), and high-frequency (HF) components; as well as the LF/HF ratio. Mean heart rate (HR) was also recorded. All recordings were performed during a 5 min resting period in the supine position under controlled conditions, with participants instructed to maintain calm, spontaneous breathing.

#### 2.3.3. The 24-Hour Holter ECG Monitoring

All participants underwent 24 h ambulatory Holter ECG monitoring. Long-term HRV analysis included the same time- and frequency-domain parameters as those used in short-term analysis, with the exception of the triangular index. Additionally, previously described Poincaré plot-derived parameters were obtained from the 24 h recordings. Recordings were performed using a three-channel Holter system (DMS ECG) with dedicated analysis software. Signal processing, RR interval extraction, artifact correction, and HRV parameter calculation were carried out using the manufacturer’s validated software, in accordance with established standards and recommendations. Preprocessing procedures, including sampling rate, artifact handling, and segment selection, were embedded within the proprietary software and were not manually modified.

### 2.4. Statistical Analysis

Continuous variables were presented as the mean ± standard deviation or median with interquartile range (IQR), as appropriate, while categorical variables were expressed as counts and percentages. Normality of continuous variables was assessed using the Kolmogorov–Smirnov test, complemented by visual inspection of histograms and Q–Q plots. Associations between Poincaré plot-derived parameters and HRV indices were evaluated using Spearman’s rank correlation coefficient due to the non-normal distribution of a substantial number of variables. Correlation strength was interpreted according to conventional criteria, with coefficients indicating weak, moderate, or strong associations [22]. To account for multiple correlation testing and reduce the risk of false-positive findings, false discovery rate (FDR) correction was applied [23]. Group comparisons between patients with normal and abnormal cardiovascular reflex test results were performed using the independent-samples *t* test for normally distributed variables and the Mann–Whitney *U* test for non-normally distributed variables. Statistical analyses were conducted using SPSS version 26.0 (IBM Corp., Armonk, NY, USA), and a two-sided *p* value < 0.05 was considered statistically significant.

## 3. Results

The demographic and clinical characteristics of the study population are summarized in Table 1. The cohort comprised 269 patients, the majority of whom were female (68%), with a mean age of 44.4 ± 14.4 years. The most prevalent clinical presentation was syncope (81%), followed by chronic fatigue syndrome (47.2%), Lyme disease (20.8%), orthostatic hypotension (18.9%), and arterial hypertension (18.2%). Other comorbidities were less frequently represented.

The Spearman correlations among Poincaré plot-derived parameters are presented in Table 2. Significant correlations were observed between multiple parameter pairs, including strong positive associations between VLI and L, SD2 and LA, as well as between L and LA. Strong positive correlations were also observed between D and SA, as well as between VAI and D and between SD1 and SA. In contrast, the indices SD1/SD2 and SD2/SD1 demonstrated a strong inverse correlation. All reported associations remained statistically significant after FDR correction.

Table 3 shows strong Spearman correlations (|ρ| ≥ 0.70) between Poincaré plot-derived parameters and long-term HRV indices. The highest positive significant correlations were observed between VLI and SDNN as well as between SD2 and SDNN. VLI also showed particularly strong positive correlations with TP and VLF. Conversely, VAI, D, SD1, and SA demonstrated strong positive correlations with pNN50. With respect to the frequency-domain measures, the largest number of Poincaré plot parameters showed strong positive correlations with TP (VLI, VAI, L, SD1, SD2 and LA) and with VLF (VLI, VAI, L, SD2, LA). The complete correlation matrix is provided in Supplementary Table S1.

**Figure 3:**
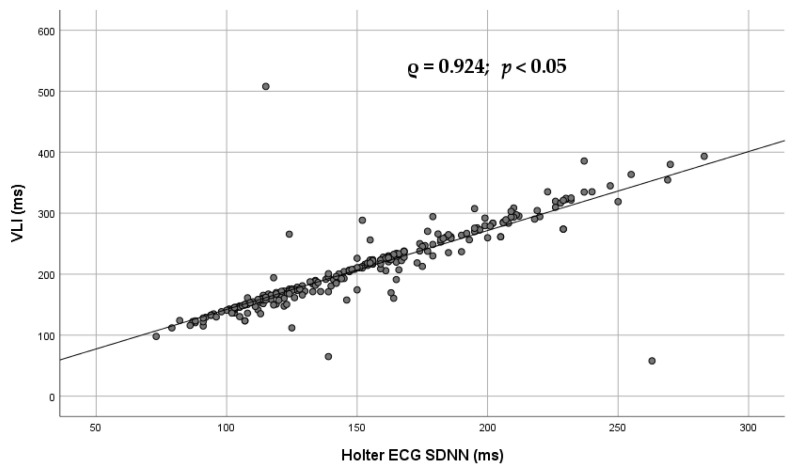
Scatter plot illustrating the Spearman association between the Poincaré plot-derived parameter VLI and long-term heart rate variability index SDNN. Each dot represents an individual subject. The Spearman rank correlation coefficient (ρ) and corresponding *p* value are shown in the panel. The *p* value was adjusted for multiple comparisons using false discovery rate (FDR) correction. The linear trend line is displayed for visualization purposes only. VLI—vector length index; SDNN—standard deviation of normal-to-normal intervals.

Figure 3 illustrates the Spearman association between the Poincaré plot-derived parameter VLI and long-term heart rate variability index SDNN.

**Figure 4:**
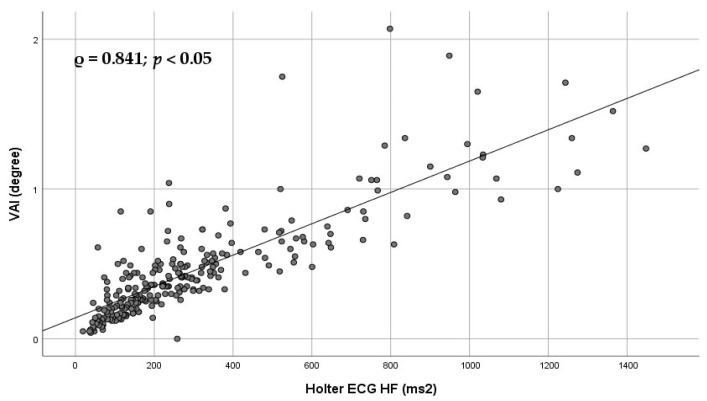
Scatter plot illustrating the Spearman association between the Poincaré plot-derived parameter VAI and HF. Each dot represents an individual subject. Spearman’s rank correlation coefficient (ρ) and corresponding *p* value are shown in the panel. The *p* value was adjusted for multiple comparisons using false discovery rate (FDR) correction. The linear trend line is displayed for visualization purposes only. VAI—vector angle index; HF—high-frequency component of heart rate variability.

Figure 4 illustrates the Spearman association between the Poincaré plot-derived parameter VAI and HF.

**Figure 5:**
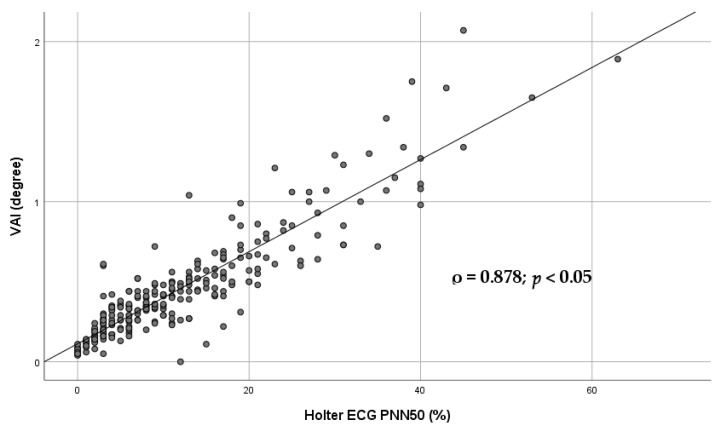
Scatter plot illustrating the Spearman association between the Poincaré plot-derived parameter VAI and pNN50. Each dot represents an individual subject. Spearman’s rank correlation coefficient (ρ) and corresponding *p* value are shown in the panel. The *p* value was adjusted for multiple comparisons using false discovery rate (FDR) correction. The linear trend line is displayed for visualization purposes only. VAI—vector angle index; pNN50—percentage of adjacent RR intervals differing by more than 50 ms.

Figure 5 illustrates the Spearman association between the Poincaré plot-derived parameter VAI and pNN50.

Supplementary Table S2 presents Spearman correlations between Poincaré plot-derived parameters and short-term HRV indices. Despite statistical significance in several comparisons, no strong correlations were detected. Only modest associations were observed, predominantly between VAI and pNN50 (Figure 3). Furthermore, most Poincaré plot-derived parameters exhibited weak inverse relationships with heart rate and the LF/HF ratio, except for SD2/SD1.

**Figure 6:**
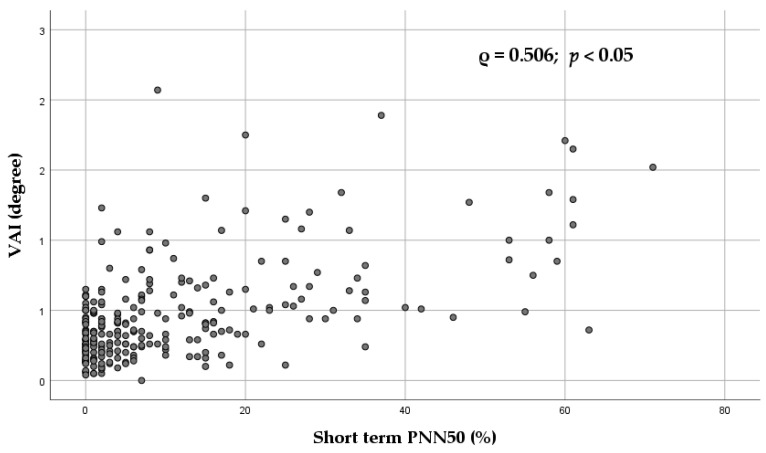
Scatter plot showing the Spearman association between VAI (Poincaré plot-derived parameter) and short-term ECG PNN50. Each point represents an individual subject. The Spearman correlation coefficient (ρ) and corresponding *p* value are displayed. *p* values were adjusted using false discovery rate (FDR) correction. The linear trend line is shown for visualization only. VAI—vector angle index; PNN50—percentage of adjacent RR intervals differing by >50 ms.

Figure 6 illustrates the Spearman association between the Poincaré plot-derived parameter VAI and short-term ECG pNN50.

Table 4 presents differences in Poincaré plot-derived parameters according to the results of the VM and HRB. Patients with abnormal VM demonstrated significantly lower values of both VLI and VAI. Similarly, VAI, SD1, LA, and SA were significantly lower in patients with abnormal HRB.

Table 5 presents differences in Poincaré plot-derived parameters according to OH status. Although the majority of parameters exhibited numerically higher mean or median values in the abnormal OH group, none of the differences reached statistical significance.

Supplementary Table S3 shows a sex-based comparison of Poincaré plot-derived parameters. Male patients showed higher values of VAI, L, SD1, SD1/SD2 and LA, respectively, which was contrary to the lower values of SD2/SD1 observed in this group when compared to female patients.

## 4. Discussion

This study integrates long-term and short-term HRV analyses with CARTs to clarify the physiological meaning of less-well-defined Poincaré plot-derived parameters in a real-world clinical population. Several parameters, particularly VLI and VAI, showed strong associations with established long-term HRV indices, whereas no strong correlations were observed with short-term HRV measures using the predefined threshold. Differences across parasympathetic reflex tests, but not orthostatic hypotension, further suggest that individual Poincaré plot parameters capture distinct aspects of autonomic regulation beyond conventional HRV metrics.

As shown in Table 2, VLI demonstrated the strongest correlations with SD2, L, and LA. Previous studies have established SD2 as a measure of long-term HRV influenced by both autonomic branches [7,16,17,18,19]. This interpretation is further supported by Table 3 and Figure 3, where VLI showed the strongest associations with SDNN and TP [7,8].

SDNN is widely regarded as a measure of overall heart rate variability and global autonomic status, reflecting the combined influence of sympathetic and parasympathetic activity. It is highly correlated with ULF, VLF, and LF band power, as well as TP [7,8]. Clinically, SDNN is considered a gold-standard parameter for risk stratification, particularly after acute myocardial infarction, but is also reduced in numerous cardiovascular and non-cardiovascular conditions [7,24,25,26,27,28,29].

Notably, VLI also exhibited a strong correlation with VLF. Although the physiological basis of VLF is not fully understood, it has been associated with adverse outcomes, inflammation, and systemic dysregulation [7]. VLF appears to reflect intrinsic cardiac nervous system activity modulated by sympathetic influences and broader regulatory mechanisms, supporting its role as an index of global and long-term autonomic organization [7].

Moreover, in the CART analyses, VLI was lower in patients with abnormal VM results but not in those with impaired HRB (Table 4). When expressed as the Valsalva ratio, the VM primarily reflects the vagal component of the baroreflex arc, whereas HRB is mediated predominantly by respiratory-driven vagal modulation of the sinus node, with contributions from the Bainbridge reflex [30,31,32,33,34]. These distinct physiological pathways may explain the differential associations observed.

Taken together, these findings suggest that VLI reflects overall and long-term autonomic organization, likely integrating both sympathetic and parasympathetic influences with a potential baroreflex-mediated parasympathetic component, rather than representing an isolated marker of a single autonomic branch.

In contrast, VAI, also previously described as a parasympathetic-related marker [15], demonstrated, among all Poincaré parameters, a strong positive correlation with SD1 and a negative correlation with the SD2/SD1 ratio (Table 2).

SD1 is considered a marker of short-term HRV and predominantly parasympathetic modulation, whereas the SD2/SD1 ratio has been proposed as a geometric surrogate of sympathovagal balance, with higher values indicating relative sympathetic predominance [7,16,17,18,19]. Furthermore, VAI demonstrated strong associations with long-term LF, pNN50 (both long and short term), and HF (Table 3; Figure 4 and Figure 5).

HF, often referred to as the respiratory band, is widely regarded as one of the principal markers of parasympathetic modulation, while pNN50 represents a classical time-domain index associated with vagal activity, although contemporary analyses more frequently emphasize RMSSD and HF [7]. With respect to LF, which was initially interpreted as reflecting sympathetic activity, current evidence suggests that it more accurately represents baroreflex-mediated autonomic regulation, with a substantial contribution from vagal modulation, particularly under resting conditions or during slow respiration, as emphasized by Shaffer et al. [7]. In addition, VAI showed strong positive correlations with TP and VLF, parameters reflecting overall heart rate variability and long-term autonomic regulation, respectively [7,8]. Unlike VLI, VAI was also significantly associated with ULF, which is thought to reflect very slow regulatory processes operating over extended time scales, including circadian rhythms and other long-term biological mechanisms [7,35]. Although the precise autonomic contributions to ULF remain incompletely understood, this finding suggests a possible link between VAI and broader, time-dependent regulatory influences rather than exclusively short-term autonomic modulation. Finally, as shown in Table 4, VAI values were significantly lower in patients with abnormal results on both the VM and HRB, respectively, which, despite involving distinct reflex pathways, are both commonly used to assess parasympathetic function [6]. Collectively, taking the CART results into consideration, the positive associations with SD1, PNN50, LF, and HF (parameters largely related to parasympathetic regulation) and the negative correlation with the SD2/SD1 ratio, VAI may predominantly reflect parasympathetic influences on cardiac autonomic control, in contrast to the more global autonomic associations observed for VLI.

The parameters L and D demonstrated strong correlations with SD2 and SD1, respectively (Table 2). L was also significantly correlated with SDNN, TP, and VLF (Table 3), a pattern similar to that observed for VLI, suggesting that L may be related to overall heart rate variability and global autonomic organization [7,8]. D, on the other hand, through its association with SD1 and pNN50, appears to be related to parasympathetic modulation, while its positive correlation with ULF (Table 3) may also indicate a link to very slow regulatory processes operating over longer time scales [7,8]. However, neither L nor D differed significantly between patients with normal and abnormal results on the VM or the HRB. Consequently, firm conclusions regarding the physiological or clinical significance of these two parameters cannot be drawn at this stage. Although the observed associations suggest a potential relation to overall autonomic status and parasympathetic modulation, further studies are required to clarify their role. 

LA, on the other hand, exhibited the same pattern of correlations as VLI, including associations with SD2, SDNN, TP, and VLF (Table 2 and Table 3), as well as a strong correlation with VLI itself (Table 2). However, in contrast to VLI, LA was reduced in patients with an abnormal HRB, but not in those with an abnormal VM. As previously noted, although both tests assess parasympathetic function, they involve distinct reflex pathways [30,31,32,33,34], with HRB predominantly reflecting respiratory-driven vagal modulation of the sinus node [35]. Taken together, LA appears, similarly to VLI, to reflect overall and long-term heart rate variability, while its selective reduction in patients with impaired HRB suggests an additional sensitivity to parasympathetic modulation mediated through respiratory-driven vagal influences on the sinus node.

A similar situation was observed for SA, which showed correlations primarily with SD1, VAI, and pNN50 (Table 2 and Table 3). Considering that VAI may reflect parasympathetic control, as well as the parasympathetic basis of SD1 and pNN50, it can be assumed that SA may also be used for the assessment of parasympathetic function [7,8,16,17,18,19]. However, unlike VAI, which was reduced in patients with both abnormal VM and abnormal HRB, SA was only significantly lower in patients with an abnormal HRB, and thus may predominantly reflect, similarly to LA, respiratory-driven vagal influences on the sinus node [35].

As shown in Supplementary Table S2, although the majority of correlations were statistically significant, they were generally weak, with the exception of the association between VAI and pNN50, which was of moderate strength (Supplementary Table S2 and Figure 6). As previously discussed, VAI may represent a marker of parasympathetic modulation. However, it is important to emphasize the different contributions of the autonomic nervous system to short-term and long-term HRV [7]. Short-term HRV is typically recorded under resting conditions in the supine position, where parasympathetic activity predominates [7]. In addition, higher levels of resting-vagally mediated HRV have been linked to better performance of executive functions, such as attention and emotional processing, mediated by the prefrontal cortex [7]. In contrast, long-term recording (of both HRV and Poincaré plot-derived parameters) reflects the system’s response to a wide range of stimuli, including stress, physical activity, orthostatic challenges, and circadian rhythms, in which sympathetic influences play a more prominent role. Therefore, short-term and long-term HRV should not be used interchangeably [7].

It is important to note the findings related to differences between patients with and without OH, as shown in Table 5. Although higher values of most Poincaré plot-derived parameters were observed in patients with abnormal OH, no statistically significant differences were detected (Table 5). OH is considered one of the principal clinical markers of sympathetic dysfunction [36]. Among all Poincaré plot parameters, only VAI showed a strong negative correlation with the SD2/SD1 ratio, where higher values indicate relative sympathetic predominance (Table 2). In addition, associations with the LF/HF ratio—both for long-term and short-term HRV measures (Table 3 and Supplementary Table S2)—were statistically significant but generally weak to modest in strength. Taken together, these findings suggest that Poincaré plot-derived parameters may have limited utility for the isolated assessment of sympathetic autonomic function. Further studies are therefore needed to better clarify their role in evaluating sympathetic regulation.

An important aspect of this study relates to specific characteristics of the study population. As noted, this cohort represents a real-world clinical population with heterogeneous diseases and comorbidities, rather than a healthy control group. This is relevant for several reasons, as different diseases may exert distinct effects on ANS, and individual patients may present with multiple coexisting diagnoses (e.g., hypertension and diabetes mellitus, or syncope and ME/CFS) [26,27,28,37,38,39,40,41,42,43,44]. In addition to the underlying disease burden, the potential influence of chronic pharmacological therapy cannot be excluded, as medications commonly used in these conditions may also affect autonomic regulation [45]. Importantly, this study was not designed as a comparative analysis between disease groups nor as an evaluation of medication effects, but rather as an exploratory, hypothesis-generating investigation in a real-world clinical setting; consequently, future studies performed in healthy populations and with controlled assessment of comorbidities and pharmacological influences are warranted to further clarify the physiological significance of Poincaré plot-derived parameters.

As shown in Supplementary Table S3 and based on our findings and previous observations on Poincaré plot-derived parameters, higher values of VLI, L, SD1, SD1/SD2, and LA in male patients may indicate greater overall autonomic variability, including parasympathetic components. In contrast, SD2/SD1, a marker of sympathovagal balance, was higher in women, suggesting relatively greater sympathetic predominance in this group. Previous studies have shown that, for the same level of HRV—especially at lower values—women tend to have higher heart rates than men, indicating that sex differences in heart rate depend on the level of autonomic variability [46]. In addition, large analyses report that women generally have lower SDNN, TP, VLF, and LF, but higher HF power, pointing to relative vagal dominance, while men tend to show relatively higher sympathetic predominance despite having lower heart rates [7]. It should also be noted that HRV declines with age, with effects varying across parameters. In older individuals, especially those >65 years, HRV may fall to levels associated with increased mortality risk. Both age and sex significantly influence HRV, and heart rate itself is also affected by these factors [47]. Importantly, this analysis represents a secondary, exploratory assessment, and male and female groups were not age-matched, which may have influenced the observed differences. Therefore, these findings should be interpreted with caution. Overall, this study is hypothesis-generating and provides initial insight into the physiological meaning of Poincaré plot-derived parameters. Future studies should further investigate these relationships, particularly with careful control of age and sex differences.

It is important to mention that both linear and nonlinear HRV analyses have important limitations in clinical practice. Conventional HRV measures are influenced by multiple external and physiological factors, particularly in long-term (Holter) recordings, including physical activity, circadian variation, and pharmacological therapy [48]. These limitations are even more pronounced for nonlinear HRV metrics, including Poincaré plot-derived parameters, which lack standardized methodology and clear physiological interpretation, thereby limiting their clinical applicability [49]. In addition, the reproducibility of certain nonlinear indices remains variable, further complicating their interpretation [50]. Taken together, these challenges highlight the need for further standardization and validation before HRV-derived parameters, particularly nonlinear ones, can be reliably applied in routine clinical practice.

### Study Limitations

Several limitations of this study should be acknowledged. Although the overall sample size was substantial, the heterogeneity of the study population—reflecting a real-world clinical setting with diverse cardiovascular and non-cardiovascular diseases and frequent comorbidities—may have introduced variability that limited disease-specific interpretation of the findings. Moreover, the potential influence of pharmacological therapy on autonomic function and HRV parameters could not be systematically assessed, as detailed medication data were not consistently available. Particular attention should be given to drugs with a known significant impact on heart rate and autonomic modulation, such as β-blockers, non-dihydropyridine calcium channel blockers, ivabradine, and related agents, which may have influenced the observed results. In addition, the absence of a healthy control group restricted the ability to define normative ranges and may have influenced the interpretation of autonomic patterns observed in this cohort. While this design allowed for an exploration of Poincaré plot-derived parameters under clinically relevant conditions, future studies performed in well-characterized healthy populations would be valuable for establishing physiological reference values. Furthermore, longitudinal studies are warranted to assess the temporal stability, progression, and potential prognostic relevance of these parameters. In addition, future research should consider integrating other nonlinear HRV methods, such as entropy-based metrics, fractal analysis, and correlation dimensions, to provide a more comprehensive assessment of autonomic regulation. Finally, although the present analysis focused on resting short- and long-term recordings, further insight may be gained by evaluating Poincaré plot-derived parameters during short, stimulus-specific intervals, such as deep breathing, VM, orthostatic challenge, or controlled pharmacological autonomic modulation using agents such as β-blockers, atropine, or scopolamine, which may help to clarify the relative sympathetic and parasympathetic contributions to these measures.

## 5. Conclusions

The Poincaré plot represents a nonlinear, geometric approach to heart rate dynamics that provides complementary information on autonomic regulation beyond conventional HRV indices. In this real-world clinical population, several Poincaré plot-derived parameters demonstrated consistent associations with established short- and long-term HRV measures, supporting their potential physiological relevance.

VLI exhibited strong correlations with parameters reflecting long-term variability and global autonomic organization, suggesting that it may correspond to an integrated measure of overall autonomic state. In contrast, VAI demonstrated a correlation pattern predominantly related to parasympathetic modulation, including strong associations with SD1, pNN50, HF, and parasympathetic cardiovascular reflex tests, indicating a closer relationship with vagal control of heart rate.

Similarly, LA showed correlation patterns comparable to those of VLI and demonstrated sensitivity to parasympathetic reflex testing, whereas SA was more closely aligned with indices of parasympathetic modulation, potentially reflecting respiratory-driven vagal influences on sinus node activity.

Overall, these findings should be interpreted within an exploratory framework. Rather than providing definitive physiological classification, the present results generate testable hypotheses regarding the autonomic significance of less-well-defined Poincaré plot-derived parameters and support further investigation using controlled, longitudinal, and stimulus-specific study designs.

## Figures and Tables

**Table 1:** Baseline characteristics of the study population.

	Study Population *N* = 269
Female (*n*, %)	183 (68%)
Age (mean ± SD)	44.4 ± 14.4
Hypertension (*n*, %)	49 (18.2%)
Hyperthyroidism (*n*, %)	2 (0.7%)
Hypothyroidism (*n*, %)	9 (3.3%)
Diabetes Mellitus (*n*, %)	3 (1.1%)
Coronary Artery Disease (*n*, %)	5 (1.9%)
Post-COVID-19 Syndrome (*n*, %)	7 (2.6%)
Chronic Fatigue Syndrome (*n*, %)	127 (47.2%)
Lyme Disease (*n*, %)	56 (20.8%)
Syncope (*n*, %)	182 (81%)
Orthostatic Hypotension (*n*, %)	51 (18.9%)

SD—standard deviation.

**Table 2:** Spearman correlation coefficients among Poincaré plot-derived parameters.

	VLI	VAI	L	D	SD1	SD2	SD1/SD2	SD2/SD1	LA	SA
VLI		0.605 *	0.840 *	0.567 *	0.671 *	0.971 *	0.152 *	−0.180 *	0.820 *	0.552 *
VAI			0.599 *	0.734 *	0.877 *	0.623 *	0.690 *	−0.712 *	0.597 *	0.722 *
L				0.610 *	0.614 *	0.868 *	0.145 *	−0.180 *	0.922 *	0.542 *
D					0.729 *	0.588 *	0.530 *	−0.548 *	0.559 *	0.912 *
SD1						0.671 *	0.795 *	−0.798 *	0.608 *	0.746 *
SD2							0.140 *	−0.176 *	0.849 *	0.556 *
SD1/SD2								−0.980 *	0.151 *	0.550 *
SD2/SD1									−0.187 *	−0.547 *
LA										0.607 *
SA										

VLI—vector length index; VAI—vector angle index; L—Poincaré length; D—Poincaré dispersion; SD—standard deviation; LA—Poincaré length; SA—Poincaré width. Values represent Spearman’s rank correlation coefficients (ρ); * *p* < 0.05 after FDR correction. Only the upper triangle of the correlation matrix is shown.

**Table 3:** Strong Spearman correlation coefficients (|ρ| ≥ 0.70) between Poincaré plot-derived parameters and long-term heart rate variability indices.

	HR	SDNN	RMSSD	PNN50	TP	ULF	VLF	LF	HF	LF/HF
VLI		0.924 *			0.755 *		0.757 *			
VAI				0.878 *	0.772 *	0.802 *	0.711 *	0.730 *	0.841 *	
L		0.846 *			0.771 *		0.774 *			
D				0.742 *		0.706 *				
SD1				0.847 *	0.725 *	0.788 *			0.753 *	
SD2		0.946 *			0.770 *		0.775 *			
SD1/SD2										
SD2/SD1										
LA		0.820 *			0.755 *		0.751 *			
SA				0.721 *	.					

VLI—vector length index; VAI—vector angle index; L—Poincaré length; D—Poincaré dispersion; SD—standard deviation; LA—Poincaré length; SA—Poincaré width; HR—heart rate; SDNN—standard deviation of RR intervals; RMSSD—root mean square differences in successive RR intervals; PNN50—percentage of adjacent RR intervals that differ > 50 ms; TP—total power; ULF—ultra-low frequency; VLF—very low frequency; LF—low frequency; HF—high Frequency. Values represent Spearman’s rank correlation coefficients (ρ).* *p* < 0.05 after FDR correction.

**Table 4:** Differences in Poincaré plot-derived parameters in the study population according to the Valsalva maneuver and heart rate response to deep breathing.

Valsalva Maneuver (VM)			
	Normal VM *N* = 218	Abnormal VM *N* = 41	*p* Value
VLI (mean ± SD)	217.4 ± 65.1	196.2 ± 65.8	0.023 ^t^
VAI (Mdn(IQR))	0.4 (0.3–0.7)	0.3 (0.2–0.5)	0.001 ^m^
L (mean ± SD)	788.1 ± 209.1	750.6 ± 173.2	0.177 ^t^
D (mean ± SD)	146.8 ± 59.2	135.2 ± 51.6	0.158 ^t^
SD1(Mdn(IQR))	27 (19–41.3)	24 (17–35)	0.072 ^m^
SD2 (mean ± SD)	214.2 ± 67.6	197.7 ± 60.2	0.066 ^t^
SD1/SD2 (Mdn(IQR))	0.1 (0.1–0.2)	0.1 (0.1–0.2)	0.271 ^m^
SD2/SD1 (Mdn(IQR))	7.8 (5.8–9.5)	8.4 (5.9–11)	0.197 ^m^
LA (Mdn(IQR))	1070 (910.5–1291.5)	1038 (883–1258)	0.130 ^m^
SA (mean ± SD)	208.5 ± 93.2	188 ± 74	0.096 ^t^
**Heart response to deep breathing (HRB)**			
	Normal HRB *N* = 42	Abnormal HRB *N* = 192	*p* value
VLI (mean ± SD)	224.3 ± 65.8	206.2 ± 65.3	0.106 ^t^
VAI (Mdn(IQR))	0.5 (0.3–0.8)	0.4 (0.2–0.5)	<0.001 ^m^
L (mean ± SD)	789.1 ± 255	764.8 ± 170.9	0.450 ^t^
D (mean ± SD)	151.1 ± 59.3	136 ± 52.4	0.100 ^t^
SD1 (Mdn(IQR))	32 (19–48.5)	24 (18–34)	0.012 ^m^
SD2 (mean ± SD)	218.8 ± 73.9	203.6 ± 63.7	0.175 ^t^
SD1/SD2 (Mdn(IQR))	0.1 (0.1–0.2)	0.1 (0.1–0.2)	0.063 ^m^
SD2/SD1 (Mdn(IQR))	7.6 (5.2–9.3)	8.1 (6.2–10.4)	0.073 ^m^
LA (Mdn(IQR))	1203 (982–1391.1)	1060 (905–1255.3)	0.023 ^m^
SA (mean ± SD)	231.8 ± 107.9	189.2 ± 73.4	0.002 ^t^

VLI–vector length index; VAI—vector angle index; L—Poincaré length; D—Poincaré dispersion; SD—standard deviation; LA—Poincaré length; SA—Poincaré width; Mdn—median; IQR—interquartile range (25–75%); ^t^—independent *t* test; ^m^—Mann–Whitney U test.

**Table 5:** Differences in Poincaré plot-derived parameters in the study population according to orthostatic hypotension.

Orthostatic Hypotension (OH)			
	Normal OH *N* = 218	Abnormal OH *N* = 41	*p* Value
VLI (mean ± SD)	198.2 (157.8–256.2)	214 (173.2–255.6)	0.417 ^m^
VAI (Mdn(IQR))	0.4 (0.3–0.6)	0.4 (0.2–0.6)	0.650 ^m^
L (mean ± SD)	774.4 ± 192.4	790 ± 146.5	0.623 ^t^
D (mean ± SD)	141.1 ± 53.9	141.9 ± 57.8	0.954 ^t^
SD1(Mdn(IQR))	24 (19–37)	26 (17.5–36.5)	0.795 ^m^
SD2 (mean ± SD)	200 (156–256)	214 (165.5–254.5)	0.615 ^m^
SD1/SD2 (Mdn(IQR))	1 (0.1–0.2)	0.1 (0.1–0.2)	0.512 ^m^
SD2/SD1 (Mdn(IQR))	7.8 (6.1–9.9)	8.1 (6.1–10.6)	0.627 ^m^
LA (Mdn(IQR))	1070 (910.5–1218)	1060 (947.5–1291.5)	0.982 ^m^
SA (mean ± SD)	187 (145–257)	187 (128.5–273)	0.815 ^m^

VLI–vector length index; VAI—vector angle index; L—Poincaré length; D—Poincaré dispersion; SD—standard deviation; LA—Poincaré length; SA—Poincaré width; Mdn—median; IQR—interquartile range (25–75%); ^t^—independent *t* test; ^m^—Mann–Whitney U test.

## Data Availability

The original contributions presented in this study are included in the article/Supplementary Materials. Further inquiries can be directed to the corresponding author.

## Supplementary Materials

The following supporting information can be downloaded at https://www.mdpi.com/article/10.3390/diagnostics16071016/s1: Table S1: Complete Spearman correlation matrix between Poincaré plot-derived parameters and long-term heart rate variability indices; Table S2: Spearman correlations between Poincaré plot-derived parameters and short-term heart rate variability indices; Table S3: Sex-based comparison of Poincaré plot-derived parameters.
