# Supplementary material for: Associations of Poincaré Plot-Derived Parameters with Heart Rate Variability and Autonomic Reflex Testing in a Real-World Clinical Population
Source: Diagnostics (Basel). 2026 Mar 27;16(7):1016. doi: 10.3390/diagnostics16071016 (PMC13072868; doi:10.3390/diagnostics16071016)
Supplement: Supplementary file 1 [file diagnostics-16-01016-s001.zip › diagnostics-4195890-supplementary.pdf]

Supplementary Table S1. Complete Spearman correlation matrix between Poincaré plot–derived parameters and long-term heart rate variability indices.

	HR	SDNN	RMSSD	PNN50	TP	ULF	VLF	LF	HF	LF/HF
VLI	-.497*	.924*	.418*	.640*	.755*	.542*	.757*	.618*	.607*	-.181*
VAI	-.315*	.621*	.607*	.878*	.772*	.802*	.711*	.730*	.841*	-.412*
L	-.526*	.846*	.401*	.617*	.771*	.487*	.774*	.650*	.580*	-.091
D	-.306*	.595*	.573*	.742*	.587*	.706*	.543*	.534*	.679*	-.434*
SD1	-.456*	.672*	.687*	.847*	.725*	.788*	.686*	.637*	.753*	-.393*
SD2	-.514*	.946*	.449*	.662*	.770*	.564*	.775*	.629*	.616*	-.181*
SD1/SD2	-.233*	.173*	.575*	.616*	.369*	.615*	.312*	.360*	.525*	-.408*
SD2/SD1	.239*	-.188*	-.594*	-.639*	-.386*	-.634*	-.323*	-.383*	-.548*	.420*
LA	-.491*	.820*	.390*	.609*	.755*	.478*	.751*	.650*	.582*	-.086
SA	-.269*	.569*	.544*	.721*	.574*	.675*	.524*	.541*	.667*	-.405*

VLI - Vector Lenght Index; VAI – Vector Angle Index; L - Poincare Lenght; D - Poincare Dispersion; SD – Standard deviation; LA – Poincare Lenght; SA - Poincare Width; HR – Heart rate; SDNN – Standard deviation of RR intervals; RMSSD – root mean square differences of succesive RR intervals; PNN50 – percentage of adjacent RR intervals that differ > 50ms; TP – Total power; ULF – Ultra low frequency; VLF – Very Low Frequency;LF – Low frequency; HF – High Frequency. Values represent Spearman's rank correlation coefficients (ϱ).* $p < 0.05$ after FDR correction.

Supplementary table S2. Spearman correlations between Poincaré plot–derived parameters and short term heart rate variability indices.

	HR	SDNN	RMSSD	PNN50	TP	VLF	LF	HF	LF/HF	TI
VLI	-.316*	.337*	.257*	.420*	.328*	.246*	.285*	.375*	-.213*	.405*
VAI	-.221*	.369*	.383*	.506*	.349*	.213*	.288*	.468*	-.298*	.254*
L	-.301*	.320*	.250*	.403*	.318*	.242*	.264*	.324*	-.150*	.406
D	-.233*	.274*	.288*	.428*	.308*	.158*	.264*	.428*	-.253*	.350*
SD1	-.283*	.362*	.357*	.493*	.321*	.200*	.264*	.415*	-.263*	.400*
SD2	-.314*	.338*	.253*	.414*	.343*	.265*	.297*	.376*	-.203*	.407*
SD1/SD2	-.154*	.254*	.317*	.357*	.146*	.038	.114*	.271*	-.220*	.214*
SD2/SD1	.163*	-.271*	-.335*	-.374*	-.169*	-.054	-.134*	-.288*	.224*	-.229*
LA	-.306*	.304*	.218*	.381*	.332*	.252*	.281*	. .346*	-.164*	.397*
SA	-.219*	.253*	.259*	.404*	.319*	.162*	.288*	.442*	-.248*	.337*

VLI - Vector Lenght Index; VAI – Vector Angle Index; L - Poincare Lenght; D - Poincare Dispersion; SD – Standard deviation; LA – Poincare Lenght; SA - Poincare Width; HR – Heart rate; SDNN – Standard deviation of RR intervals; RMSSD – root mean square differences of succesive RR intervals; PNN50 – percentage of adjacent RR intervals that differ > 50ms; TP – Total power; ULF – Ultra low frequency; VLF – Very Low Frequency;LF – Low frequency; HF – High Frequency. Values represent Spearman's rank correlation coefficients (ϱ).* $p < 0.05$ after FDR correction.

Supplementary table S3. Sex-based comparison of Poincaré plot–derived parameters.

	Male N = 86	Female N = 183	P value
VLI (ms) (mean ± SD)	219.3 ± 64.4	205.4 ± 65.5	.104^{t}
VAI (degree) (Mdn(IQR))	.48 (.33 - .54)	.35 (.20 -.54)	.001^{m}
L (ms) (mean ± SD)	833 ± 191.1	750.4 ± 177.9	.001^{t}
D (ms) (mean ± SD)	149.3 ± 49	137.2 ± 55.8	.087^{t}
SD1 (ms) (Mdn(IQR))	28.5 (22 – 39)	22 (16-36)	< .001^{m}
SD2 (ms) (mean ± SD)	218.4 ± 64.6	203.4 ± 65.4	.079^{t}
SD1/SD2 (Mdn(IQR))	.14 (.12 - .17)	.12 (.09 - .16)	.001^{m}
SD2/SD1 (ms) (mean ± SD)	7.35 ± 2.5	9 ± 4.7	.002^{t}
LA (ms) (Mdn(IQR))	1164 (968.3 – 1382.8)	1043.5 (905 – 1237)	.002^{m}
SA (ms) (mean ± SD)	207.3 ± 83.4	195.8 ± 86	.300^{t}

VLI - Vector Length Index; VAI – Vector Angle Index; L - Poincare Length; D - Poincare Dispersion; SD – Standard deviation; LA – Poincare Length; SA - Poincare Width; ms – miliseconds; Mdn – Median; IQR – Interquartile range (25-75%); ^{t} – Independent T test; ^{m} – Mann Withney U test.
